# Estimating gestational age using placental thickness and fetal weight

**DOI:** 10.6026/973206300210205

**Published:** 2025-02-28

**Authors:** Durgesh Singh, Neha Gupta, Pankaj Singh, Sanjay Singh, Shashi Prabha Singh, Shri Lal Raghudev Ram Singh

**Affiliations:** 1 Department of Anatomy, Maa Vindhyavasini Autonomous State Medical College, Mirzapur, Uttar Pradesh, India; 2 Department of Anatomy, SMMH Medical College, Saharanpur, Uttar Pradesh, India; 3 Department of Anatomy, Saraswati Medical College, Unnao, Uttar Pradesh, India; 4 Department of Anatomy, Rajshree Medical College Bareilly, Uttar Pradesh, India; 5 Department of Biochemistry, Maa Vindhyavasini Autonomous State Medical College, Mirzapur, Uttar Pradesh, India; 6 Department of Applied Mathematics The University of Waterloo, Canada

**Keywords:** Gestational age, fetus weight, placenta thickness, ultra-sonographic

## Abstract

The correlation between placental thickness, gestational age and fetal weight across different stages of pregnancy is of interest. Hence, 187 women (11-40 weeks gestation) at Maa Vindhyavasini Autonomous State Medical College and SMMH Medical College in India participated in this study. Results showed that mean placental thickness increased with gestational age (2.083 cm at 11-20 weeks, 2.623 cm at 20-30 weeks and 3.29 cm at 30-40 weeks), while fetal weight also increased (172.05g, 746.67g and 2584.49g, respectively). A strong positive correlation was found between gestational age and both fetal weight and placental thickness, except during early pregnancy (11-21 weeks), with statistical significance at the 5% level. Thus, the correlation between placental thickness, fetal weight and gestational age in 187 pregnant women, showing strong positive correlations, especially in later stages of pregnancy is reported.

## Background:

Thickness of placenta of pregnant women appears to be a favourable parameter for estimation of gestational age and fetus weight. From the past studies, it was observed that there is good positive correlation between thickness of placenta and gestational age of women. The measurement of thickness of placenta is a crucial parameter for the estimation of gestational age especially for the 11-20 weeks, 21-30 weeks and 31-40 weeks, where the exact duration of pregnancy is not known and sometimes other sonographic parameters is also become unreliable. In a past study Hafner *et al.* (1998) [1] have reported in their study has been recognized foetuses at risk of growth restriction using second trimester placental volumes measured by three-dimensional ultrasound while in another study conducted by Habib (2002) [2] reported that ultrasonographic measurement of placental diameter and thickness is of prognostic value in identifying the subsequent occurrence of fetal growth restriction. Dare *et al.* (1990) [3] in a study mentioned that fetal weight estimation is an important aspect of obstetric management and is variously carried out by tactile assessment of fetal size, maternal self-estimation [Chauhan *et al.* (1992), Baum *et al.* (2002)] [4- 5], birth-weight prediction equation Dare *et al.* (1990) [3] and using algorithm derived from maternal and pregnancy-specific characteristics Nahum (2007) [6]. Therefore, it is of interest to know mean scores of placenta thickness and weight of fetus according to their different gestation periods which is categories in three groups specially (11-20 weeks), (21-30 weeks) and (31-40 weeks), to establish the correlation between thickness of placenta as well as fetus weight and gestation ages of women.

## Methods and Materials:

This study was conducted at the Anatomy, Obstetrics & Gynecology and Radiology Department, Maa Vindhyavasini Autonomous State Medical College, Mirzapur, Uttar Pradesh, India and collaboration with SMMH Medical College Saharanpur UP India. The ethical approval was done from the institutional Ethics committee. A total of 187 samples out of 200 were identified for the study purpose. The pregnant women were asked to lay in the supine the thickness of placenta and measured at level of cord insertion. Transducer has been oriented perpendicular to scan both chorionic and basal plates. The measurements of placental thickness have been recorded in centimetres. Each case has been followed up to thrice *i.e.* three times evaluations have been recorded for each case. Ultrasound machine GE Voluson E6 convex probe 3-5 MHz frequency was used and measure placental thickness in Antero-posterior dimension.

## Inclusion criteria:

[1] Patient who was ready to involve in study after informed consent of study.

[2] Women with 11 to 40 weeks pregnancy.

[3] Regular menstrual history before current pregnancy.

## Exclusion criteria:

[1] Maternal disease - thyroid disease

[2] Gestational diabetes

[3] Hypertension

[4] Anemia

[5] Placenta prevails, placental anomalies and poor visualization of placenta

[6] Multiple pregnancies

[7] Last menstrual period not known or irregular menstrual period

## Statistical analysis:

The data was analyzed using SPSS version 26.0 software. Univariate and bivariate tables were employed to present the findings. Pearson correlation and scatter plots were used to examine the relationship between gestational age, placental thickness and fetal weight. Statistical significance was assessed at the 5% level.

## Results:

## Gestational age:

In this section, univariate, bivariate and scatter diagram as well as Person correlation coefficient have been used to describe for presentation of the data. Figure 1 - (see PDF) shows that age wise percentage distribution of respondents. Most of the respondents (31%) were belong to age group (< 22) years and same percentage were observed for age group (25-30) years. Table 1 shows that gestational age (11-40 weeks) wise distribution of respondents. Majority of respondents (77.5%) were belonged to gestation age (15-21) week. In case of gestational age (21-31weeks) wise distribution of respondents, majority of respondents (71.7%) were belonged to gestation age (21-24) weeks and followed by 28.3 % for the gestation age (24-31) weeks. Similarly gestational age (31-41 weeks) wise distribution of respondents, majority of respondents (76.5%) were belonged to gestation age (35-41) weeks and followed by 23.5 % for the gestation age (31-35) weeks. Table 2 indicates that descriptive statistics of thickness of placenta and weight of fetus according different gestation age of respondents. Mean scores of thicknesses of placenta were found 2.08cm, 2.62 cm and 3.29 cm for (11-20), (21-30) and (31-40) weeks respectively. Mean scores of gestation age were found 16 weeks & 6 days, 24 weeks & 5 days and 35 weeks & one days for (11-20), (21-30) and (31-40) weeks of gestation ages respectively. Mean scores of weights of fetus were found 83.95 gm, 367gm and 543. 40 gm for (11-20), (21-30) and (31-40) weeks of gestation ages respectively. Table 3 shows that correlation between Gestation age, thickness of Placenta and weight of fetus (11-20 weeks). It was found that there is very strong positive correlation (0.937) between gestation age and weight of fetus and it was statistically significant at 5 % level of significance whereas correlation between thickness of placenta and gestation age was found almost zero and negative correlation (-0.073). Almost same correlation coefficient (-0.038) was found between thickness of placenta and weight of fetus and it was insignificant at 5 % level of significance. It may be concluded that there was no significant relation between thickness of placenta and gestation age specially (11-20) weeks of respondents. Table 4 shows that correlation between Gestation age, thickness of Placenta and weight of fetus (21-31 weeks). It was found that there is very strong positive correlation (0.944) between gestation age and weight of fetus and it was statistically significant at 5 % level of significance whereas correlation between thickness of placenta and gestation age was also found positive moderate relation (0.437) and it was found significant at 5% level of significance. Correlation between thickness of placenta and weight of fetus was also found average positive relation (0.430) and it was significant at 5 % level of significance. Table 5 shows that correlation between Gestation age, thickness of Placenta and weight of fetus (31-41 weeks). It was found that there is very strong positive correlation (0.975) between gestation age and weight of fetus and it was significant at 5 % level of significance whereas correlation between thickness of placenta and gestation age was also found positive average relation (0.519) and almost same correlation (0.521) was observed between thickness of placenta and weight of fetus it was found significant at 5% level of significance. It was observed from the above Table 4, Table 5 that correlation among gestation age, thickness of placenta and weight of fetus were increasing as increased of gestation of age except for the gestation age (11-20) weeks of respondents.

## Discussion:

Placental thickness and fetal weight were recorded across different gestational age groups, specifically 11-20, 20-30 and 30-40 weeks. The discussion in this section is based on the analysis of data and results. Most respondents (31%) fell into the age groups of under 22 years and 25-30 years. The mean placental thickness and fetal weight for the gestational age groups 11-20, 20-30 and 30-40 weeks were 2.083 ± 0.56 cm, 2.623 ± 0.34 cm and 3.29 ± 0.26 cm and 172.05 ± 83.95 g, 746.67 ± 367.22 g and 2584.49 ± 543.41 g, respectively. A strong positive correlation was observed between fetal weight and gestational age. For gestational age 11-20 weeks, the correlation between gestational age and fetal weight was very strong (0.937) and statistically significant at the 5% level. This finding aligns with a similar study by Karthikeyan *et al.* (2012) [7]. However, no significant correlation was observed between placental thickness and fetal weight for this gestational age group. In a study by Banik *et al.* (2022) [8], an ultra-sonographic analysis of placental maturity showed that placental thickness was roughly equivalent to gestational age. For gestational age 20-30 weeks, a strong positive correlation (0.944) was found between gestational age and fetal weight, which was statistically significant at the 5% level. The correlation between placental thickness and gestational age was moderate (0.437), also significant at the 5% level. These findings are consistent with previous studies, such as those by Karthikeyan *et al.* (2012) [7], Larcher *et al.* (2023) [9] and Rawal *et al.* (2024) [10]. For gestational age 31-40 weeks, a strong positive correlation (0.975) was found between gestational age and fetal weight, significant at the 5% level. The correlation between placental thickness and gestational age was good (0.519), with a similar correlation (0.521) observed between placental thickness and fetal weight, both significant at the 5% level. These results are consistent with a study by Mathai *et al.* (2013) [11]. Vinchurkar *et al.* (2023) Placental thickness is closely associated with fetal weight during gestation. Regular measurement of placental thickness can assist in estimating gestational age and identifying babies with intrauterine growth restriction (small for gestational age) [12]. Table 3, Table 4 and Table 5 the correlations between gestational age, placental thickness and fetal weight increased with gestational age, except for the 11-20-week group. This variation may be attributed to data collection during the COVID-19 pandemic. The study was based on a small sample and placental thickness and fetal weight were measured for gestational ages ranging from 11-40 weeks. A larger sample size with a longitudinal study design would provide more accurate measurements of placental thickness and could help predict the exact delivery date for pregnant women. There is a significant positive correlation between placental thickness and fetal weight, making the measurement of placental thickness at the umbilical cord insertion site a dependable sonographic predictor of fetal weight [13].

## Conclusion:

A strong, positive correlation was observed between placental thickness and gestational age, with placental thickness consistently increasing throughout pregnancy. Fetal weight also followed a predictable growth pattern, aligning with established gestational age markers. Combining these two measurements offers a non-invasive, effective method for gestational age estimation, especially in cases where traditional methods, such as last menstrual period or early pregnancy ultrasound, are unavailable or unreliable. Thus, the strong correlation between placental thickness and fetal weight provides promising alternatives for accurate gestational age estimation, potentially improving prenatal care in challenging clinical situations.

## Figures and Tables

**Table 1:** Gestational age (11-40 weeks) wise distribution of respondents

**Gestational age group**	**Gestational age (week)**	**Frequency**	**Percent**
1^st^ group (11-20) weeks	< 15	42	22.5
	15-21	145	77.5
2^nd^ group (21-30) weeks	21-24	134	71.7
	24-31	53	28.3
3^rd^ group (31-40) weeks	31-35	44	23.5
	35-41	143	76.5
Total		187	100

**Table 2:** Descriptive statistics of fetus measurement and gestational ages of the respondents

**Gestational age group**	**Variable**	**Minimum**	**Maximum**	**Mean**	**St. Deviation**
11-40 weeks	Gestation age	12.14	21	16.5844	2.209
	Placenta thickness	1.20cm.	3.20cm	2.083 cm	.5666 cm
	Fetus weight	54.00gm	412.0gm	172.0588 gm.	83.9531gm
21-30 weeks	Gestation age	20.14	30.43	24.4905	3.007
	Placenta thickness	2.00cm	4.20cm	2.622cm.	.3465cm
	Fetus weight	21.00gm	1589.0gm	746.6738gm.	367.2240gm
31-40	Gestation age	28.57	40.57	35.1245	2.3331
Weeks	Placenta thickness	2.00cm	3.91cm	3.290cm.	.2593cm
	Fetus weight	1230gm	4101gm	2584.49 gm.	543.406gm

**Table 3:** Correlation between gestation age, thickness of placenta and weight of fetus (11-20 weeks)

**Gestational age (11-20 weeks)**		**Gestation age**	**Thickness of Placenta**	**Weight of foetus**
Gestation age	Pearson Correlation	1	-0.073	.937**
	Sig. (2-tailed)		0.318	0
Thickness of Placenta	Pearson Correlation	-0.073	1	-0.038
	Sig. (2-tailed)	0.318		0.607
eight of fetus	Pearson Correlation	.937**	-0.038	1
	Sig. (2-tailed)	0	0.607	
	N	187	187	187
** Correlation is significant at the 0.01 level (2-tailed)

**Table 4:** Correlation between gestation age, thickness of placenta and weight of fetus (21-30 weeks)

**Gestational Age (21-30 weeks)**		**Gestation age**	**Thickness of Placenta**	**Weight of fetus**
Gestation age	Pearson Correlation	1	.437**	.944**
	Sig. (2-tailed)		0	0
Thickness of Placenta	Pearson Correlation	.437**	1	.430**
	Sig. (2-tailed)	0		0
Weight of fetus	Pearson Correlation	.944**	.430**	1
	Sig. (2-tailed)	0	0	
	N	187	187	187

**Table 5:** Correlation between Gestation age, thickness of Placenta and weight of fetus (31-40 weeks)

**Gestational Age (31-40 weeks)**		**Gestation age**	**Thickness of Placenta**	Weight of fetus .975**
Gestation age	Pearson Correlation	1	.519**	
	Sig. (2-tailed)		0	0
Thickness of Placenta	Pearson Correlation	.519**	1	.521**
	Sig. (2-tailed)	0		0
Weight of fetus	Pearson Correlation	.975**	.521**	1
	Sig. (2-tailed)	0	0	
	N	187	187	187
